# Antimicrobial Properties of Hive Products and Their Potential Applications in Human and Veterinary Medicine

**DOI:** 10.3390/antibiotics14020172

**Published:** 2025-02-10

**Authors:** Roberto Bava, Claudio Puteo, Renato Lombardi, Giuseppe Garcea, Carmine Lupia, Angelica Spano, Giovanna Liguori, Ernesto Palma, Domenico Britti, Fabio Castagna

**Affiliations:** 1 Department of Health Sciences, University of Catanzaro Magna Græcia, 88054 Catanzaro, Italy; roberto.bava@unicz.it (R.B.); palma@unicz.it (E.P.); britti@unicz.it (D.B.); 2 Department of Clinical and Experimental Medicine, University of Foggia, 71121 Foggia, Italy; claudio_puteo.607989@unifg.it; 3 Local Health Autorithy (ASL), 71121 Foggia, Italy; renato.lombardi@aslfg.it (R.L.); giovanna.liguori@aslfg.it (G.L.); 4 Catanzaro Veterinary Centre (CeVeCa), 88100 Catanzaro, Italy; garceavet@gmail.com; 5 Mediterranean Ethnobotanical Conservatory, 88054 Catanzaro, Italy; studiolupiacarmine@libero.it; 6 Department of Pharmacy-Pharmaceutical Sciences, University of Bari, 70121 Bari, Italy; angelicaspano98@gmail.com

**Keywords:** hive products, antimicrobial activity, veterinary medicine, honey, propolis, one health

## Abstract

Hive products, encompassing honey, propolis, bee venom, royal jelly, and pollen, are recognized for their antimicrobial and therapeutic properties. This review examines their chemical composition, explores their mechanisms of action, and discusses their potential applications in both human and veterinary medicine, particularly in addressing the challenge of antimicrobial resistance. This study utilized a comprehensive literature search strategy, gathering data from Google Scholar, MEDLINE PubMed, SciELO, and SCOPUS databases. Relevant search terms were employed to ensure a thorough retrieval of the pertinent literature. Honey, rich in bioactive compounds such as hydrogen peroxide and methylglyoxal, effectively disrupts biofilms and combats multi-drug-resistant pathogens, showing promise in treating a range of infections. Propolis, with its flavonoids and phenolic acids, demonstrates synergistic effects when used in conjunction with antibiotics. Bee venom, particularly its component melittin, exhibits antibacterial and immunomodulatory properties, although further research is needed to address toxicity concerns. Pollen and royal jelly demonstrate broad-spectrum antimicrobial activity, which is particularly relevant to animal health. Existing pre-clinical and clinical data support the therapeutic potential of these hive products. Hive products represent a vast and largely untapped natural resource for combating antimicrobial resistance and developing sustainable therapies, particularly in the field of veterinary medicine. However, challenges remain due to the inherent variability in their composition and the lack of standardized protocols for their preparation and application. Further research is essential to fully elucidate their mechanisms of action, optimize formulations for enhanced efficacy, and establish standardized protocols to ensure their safe and effective clinical use.

## 1. Introduction

Honey, propolis, royal jelly (RJ), beeswax, bee venom (BV), pollen, and honey-fermented compounds are natural products that have been utilized in medicine since ancient times. Despite significant chemical differences, bee products share common properties, including antibacterial, antifungal, antiviral, antiparasitic, anti-inflammatory, antiproliferative, and antioxidant activities [1,2]. Ancient civilizations, such as the Egyptian and Greek, had already recognized the healing properties of bee products, employing them to promote wound healing, stimulate expectoration, strengthen the immune system, and protect the body from infections [3]. However, over time, with the advent of modern medicine and the introduction of synthetic drugs, the use of natural products, including bee-derived substances, gradually diminished in many Western societies. This shift was largely driven by the introduction of antibiotics, one of the most revolutionary discoveries in modern medicine. Antibiotics, due to their ability to rapidly and effectively treat a wide range of infections, quickly became a cornerstone of modern medical systems [4].

Today, however, we are witnessing a significant reversal of this trend. An increasing number of bacteria are becoming resistant to multiple antibiotics currently in use, leading to the rise of multidrug-resistant pathogens, which pose a serious challenge to healthcare systems worldwide [5,6,7,8].

In this context, bee products, with their potential antimicrobial activity and low risk of inducing resistance, are receiving renewed attention as promising allies in the fight against multidrug-resistant pathogens, as evidenced by numerous studies [9,10,11,12]. For instance, honey has proven particularly effective in the treatment of wounds and burns that are resistant to conventional therapies [9,10,11,12,13,14,15,16,17]. Among the various types of honey, Manuka honey, produced from the nectar of *Leptospermum scoparium* shrub native to New Zealand and Southeastern Australia, stands out for its exceptional effectiveness. This honey owes its antibacterial properties to the high concentration of methylglyoxal (MGO), considered one of the key components with antibacterial properties found in Manuka honey [18].

Similarly, components of RJ, such as trans-10-hydroxy-2-decenoic acid, royalisin, its main proteins, and their cleavage products like jelleine [19,20], as well as glucose oxidase, contribute to antimicrobial activity against bacteria, fungi, and viruses [21,22]. BV itself, containing its main components, melittin and PLA2, has demonstrated antibacterial properties by damaging and ultimately inducing lysis of the bacterial cell wall [23,24]. The caffeic acid phenethyl ester present in propolis also exhibits antibacterial activity [25]. Moreover, pollen and beeswax have been reported to possess antimicrobial properties as well [26,27].

**Figure 1:**
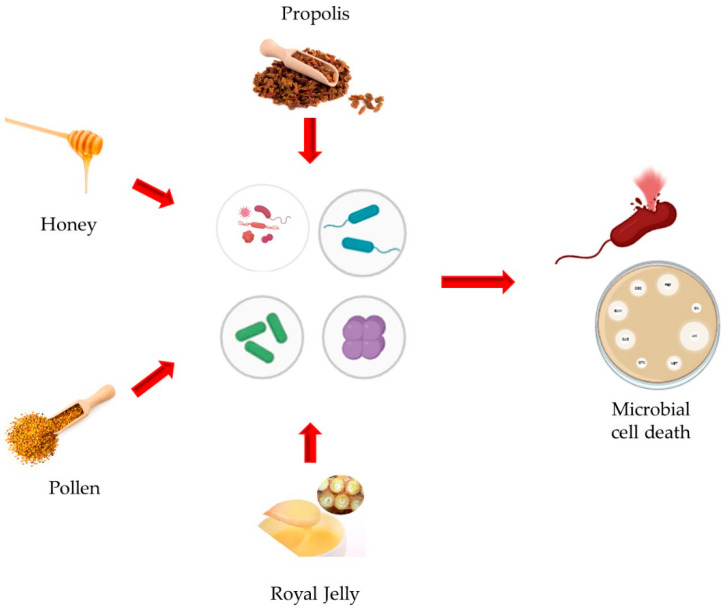
Bee products as a source of natural antimicrobials for veterinary applications.

All bee products represent an extraordinary natural resource with a broad spectrum of therapeutic properties, as summarized in Figure 1. The increasing need for alternatives to traditional antibiotics in response to the phenomenon of bacterial resistance underscores the importance of further exploring the knowledge and potential use of these natural substances in both medical and veterinary contexts.

The aim of this work is to provide a comprehensive overview of the primary bee products, analyzing their chemical characteristics, biological properties, and practical applications, with a particular focus on their use in veterinary medicine. The growing need for alternative antimicrobial strategies underscores the importance of further research into these promising natural resources.

## 2. Honey’s Antimicrobial Action: Bioactive Components and Therapeutic Potential Against Multidrug-Resistant Pathogens

Honey is a complex food substance composed of over 200 compounds, including sugars, water, proteins, vitamins, minerals, phenolic compounds, and plant derivatives [28,29,30]. Carbohydrates, primarily glucose and fructose, account for more than 80% of honey’s composition [31]. These, combined with a low water content (13–23%), create a hyperosmotic environment that inhibits microbial growth [31,32]. Furthermore, honey’s acidic pH (3.2–4.5) and high osmotic pressure further contribute to its antimicrobial activity [33].

One of honey’s key antimicrobial components is hydrogen peroxide, generated by the enzyme glucose oxidase during the oxidation of glucose [33]. This oxidative agent irreversibly damages the DNA and cell membranes of microorganisms [34,35,36]. However, honey such as Manuka exhibits non-peroxide antimicrobial activity, which is attributed to MGO, a compound derived from dihydroxyacetone found in the nectar of Leptospermum species [37,38]. MGO interferes with bacterial protein structures, causing membrane damage and reducing cellular motility [18].

Phenolic compounds, including flavonoids and phenolic acids, are another significant class of antimicrobial components. These compounds, particularly abundant in Tualang honey, are derived from plant nectar and are known to inhibit the growth of Gram-negative bacteria such as *Klebsiella pneumoniae*, *Pseudomonas aeruginosa*, and *Acinetobacter baumannii* [39,40]. Additionally, their antioxidant properties contribute to the stability of hydrogen peroxide in honey [30,41].

Defensin-1, or royalisin, is an antimicrobial peptide produced by the hypopharyngeal glands of bees, playing a crucial role in the innate immune response of bees. It exhibits activity against fungi, yeasts, protozoa, and both Gram-positive and Gram-negative bacteria [42]. In vitro studies have demonstrated that royalisin is particularly effective against Gram-positive bacteria, such as *Bacillus subtilis* and *Staphylococcus aureus*, by creating pores in bacterial cell membranes, leading to cell death [33,43,44].

Finally, honey’s antimicrobial activity is enhanced by its complex composition, which includes organic acids, enzymes, and other secondary metabolites. These elements act synergistically, making honey a multifunctional antimicrobial agent resistant to the development of bacterial resistance phenomena [45].

The use of honey, especially varieties with high concentrations of bioactive compounds like Manuka and Tualang honeys, represents a promising opportunity for alternative antimicrobial treatments, particularly against multi-resistant pathogens.

In a study by Brown et al., after confirming the compositional properties of Manuka honey, it was tested against *Staphylococcus pseudintermedius*, a Gram-positive opportunistic pathogen that causes severe infections in animals and can be transmitted to other species, including humans. The results demonstrated that Manuka honey was not only effective against multi-drug-resistant strains of *Staphylococcus pseudintermedius* but could also be used in combination with antibiotics to significantly enhance the healing process [46].

Another study evaluated the combination of Manuka honey with various antibiotics (Gentamicin—Gen, Vancomycin—Van, and Rifampicin—Rif) against three different strains of *Staphylococcus*: *S. aureus*; *S. epidermidis*; and *S. lugdunensis*, including their small colony variants (SCV)s. The combination of honey and antibiotics showed additive and partially synergistic effects, significantly reducing the minimum inhibitory concentration (MIC) of the antibiotics. Among the most significant results, the honey–Rifampicin and honey–Vancomycin combinations reduced the MIC of *S. aureus* by threefold, while the honey–Gentamicin combination showed an additive effect, reducing the MIC twofold. Gentamicin was the only antibiotic that produced resistance in the SCVs of the three tested *Staphylococcus* strains. However, the addition of honey completely suppressed SCV formation in vitro [47].

In another study, it was shown that a 10% concentration of honey can inhibit biofilm formation by oral bacteria such as *Streptococcus mutans*, suggesting a potential role for honey in reducing oral pathogens found in dental plaque [48]. Additionally, honey proved effective against biofilms of methicillin-sensitive *Staphylococcus aureus* (MSSA), methicillin-resistant *Staphylococcus aureus* (MRSA), and *Pseudomonas aeruginosa*, with bactericidal rates of 63–82%, 73–83%, and 91%, respectively, surpassing the effectiveness of many commonly used antibiotics [49].

Regarding clinical studies on honey use, Blaser et al. documented complete healing in seven consecutive patients with infected or MRSA-colonized wounds. In contrast, the antiseptics and antibiotics previously used had failed to resolve the symptoms associated with the infection [50].

An additional study tested honey as an alternative to standard fluconazole treatment for recurrent vulvovaginal candidiasis (RVVC). Due to its multiple antimicrobial, antioxidant, and anti-inflammatory activities, honey was effective against resistant strains of *Candida* and established biofilms. Clinical studies demonstrated that honey significantly reduced symptoms (itching, discharge, burning) and showed efficacy comparable to or exceeding traditional treatments like clotrimazole and fluconazole in preventing recurrences [51].

However, the unpredictability of honey’s constituents, reflected in the lack of standardization of its antimicrobial activity, hampers its widespread use in clinical practice. Factors such as the type of bee that collects the honey, the plants from which it is gathered, the climate, location, and storage conditions all influence the chemical composition of honey [18,28,29,30,31,32,33,34,35,36,37,38,39,40,41,42,43,44,45,46,47,48,49,50,51,52].

### Advancing Veterinary Medicine: Using Bee Honey to Support Animal Health and Treat Infections

Honey is not only a potent ally for human health, but it also represents a valuable resource in veterinary medicine. Its antimicrobial, anti-inflammatory, and wound-healing properties have proven effective in treating various infectious conditions and supporting wound healing in animals. This notion is supported by numerous pre-clinical and clinical studies conducted in animals over the years.

In South Africa, the effectiveness of medical-grade honey for wound care in rhinoceroses has been studied. The cohort involved seven rhinoceroses with wounds of different etiologies, including gunshot injuries and cuts. The results highlighted rapid healing in all cases, with infections resolving where present. Notably, Case 2 involved a rhinoceros shot with a firearm. An antibiogram revealed an ongoing infection with *Enterococcus faecalis* and *Aeromonas hydrophila*, which were sensitive to florfenicol, ceftiofur, and enrofloxacin. However, the application of medical-grade honey demonstrated strong antimicrobial activity, rendering the aforementioned antibiotics unnecessary for treatment [53].

A similar outcome was observed in a pre-clinical study involving 40 rabbits. The rabbits were randomly divided into four groups (A, B, C, and D). Using aseptic surgical techniques, a 3 cm incision was made on the left thigh of each rabbit. Wounds from five rabbits in each group were treated twice daily with a topical application of 5 mL of pure honey. The other half of each group served as controls and was left untreated. The results showed that the lesions treated with honey exhibited less edema and necrosis, along with better epithelialization and wound contraction. These findings suggest that honey, when applied topically to skin wounds, accelerates the healing process and demonstrates significant properties, making it an ideal dressing for skin wounds [54].

Honey has also been proven to be an excellent medical and antimicrobial agent in horses. In a randomized, open-label, prospective clinical study, the healing time and infection incidence of wounds were examined in 127 horses. Sixty-nine horses were treated with medical honey, while the remaining 56 horses served as the control group. Horses treated with medical honey had a higher likelihood of achieving complete healing (*p* = 0.006) and were less likely to exhibit signs of infection (*p* = 0.007) compared to the control group [55].

The use of honey in veterinary medicine not only provides a natural and effective solution for wound treatment but also presents a promising opportunity to address a wide range of animal diseases, both in domestic and wild animals. A recent pre-clinical study in rats demonstrated how two different types of Saudi honey, Wadi and Talh honey, exerted protective effects against ulcers induced by indomethacin. The results showed a significant reduction in ulcer indices and protection of the gastric mucosa. Following treatment, low levels of inflammatory cytokines (TNF-α, IL-10) were recorded, and restoration of antioxidant function was noted, as indicated by evaluations of Glutathione (GSH), Superoxide Dismutase (SOD), and Glutathione Peroxidase (GPx) [56]. More recent studies have investigated the antimicrobial and anti-inflammatory activity of Manuka honey in three cases of synovial septicemia in horses. These clinical cases document the immediate postoperative introduction of intra-synovial honey following treatment for synovial septicemia. In all three cases, the sterile Manuka honey applied within the inflamed synovial cavities was found to be safe. The horses recovered and returned to walking. The use of honey has shown promise as a potential adjunctive therapy following conventional surgical treatment for septic arthritis [57].

The studies presented highlight excellent results across various animal species. However, the limitations of this scientific literature should be considered. Many studies have a small number of enrolled animals, necessitating further investigation into the reproducibility of the findings. Additionally, the lack of full standardization in treatment protocols, including variability in the types of honey used, complicates the introduction of honey into routine veterinary clinical practice. Therefore, while the available data are promising, further studies with larger sample sizes and rigorous methodologies are required to consolidate and validate the use of honey in veterinary medicine.

Table 1 summarizes the key compounds, antimicrobial properties, and veterinary applications of various honey.

## 3. Bee Venom: Bioactive Components and Therapeutic Potential

BV is a widely recognized toxin secreted by the venom glands of worker bees as a defense mechanism [58]. While toxic to predators, BV has been used for medicinal purposes since ancient Egypt (4000 BC) and remains a prominent part of traditional medicine for treating various ailments [59]. Traditional therapy involves live bees being applied to specific acupuncture points in affected areas. The effectiveness of BV peaks when collected directly from a live bee during late spring to early autumn, a period when bees have access to abundant pollen sources, facilitating the production of particularly potent venom. In contrast, venom produced during the winter months is generally considered less powerful [60].

BV is a complex mixture of pharmacologically active peptides and enzymes, although its specific composition may vary among different bee species.

The venom of *Apis mellifera* is a clear, odorless liquid composed of 88% water and only 0.1 µg of venom per unit volume [23] and rich in peptides [61]. For instance, melittin (Mel), one of the most significant components, contains 26 amino acids organized in a complex structure. Mel constitutes approximately 40–50% of the venom’s weight and exhibits amphipathic properties. Biological activity, as reflected in mel content, is a crucial determinant of BV quality [62]. Another peptide present in smaller amounts is apamin, which contains 18 amino acids and is used to treat certain neurological disorders, such as Parkinson’s disease [63]. Among the complete enzymatic profile of BV, phospholipase A2 (PLA2) is recognized as one of the primary toxins, constituting up to 12% of the venom. This enzyme damages the phospholipids in cellular membranes, leading to significant cellular injury [62].

Numerous studies have demonstrated the bacteriostatic and bactericidal properties of BV against both Gram-negative and Gram-positive bacteria [64,65]. Issam AL-Ani et al. tested the antibacterial activity of *Apis mellifera* BV and isolated Mel against 51 bacterial strains, with strong activity against MRSA and VRE, either alone or in combination with antibiotics such as vancomycin, oxacillin, and amikacin. In all cases, both BV and Mel exhibited antibacterial activity, enhancing the bactericidal effect when used alongside antibiotics [64].

However, Gram-negative strains proved more resistant to BV. This resistance can be attributed to the nature of the bacterial cell membrane. The outer membrane of Gram-negative bacteria contains lipopolysaccharides (LPS) that hinder the penetration of Mel, whereas Gram-positive bacteria lack LPS, allowing Mel to more easily penetrate the cell membrane, form pores, and increase cellular permeability [64,65,66,67]. BV has also proven effective in reducing biofilm formation in foodborne pathogens such as *Salmonella*. In a study involving 16 poultry-derived strains, BV reduced biofilm formation in 47% of cases and improved bacterial motility, with a minimum inhibitory concentration (MIC) ranging from 256 to 1024 µg/mL [68]. Other studies have confirmed that BV is effective against bacteria such as *S. aureus* and *E. coli*, preventing regrowth at high concentrations. For Gram-positive bacteria such as *B. subtilis*, the MIC ranged from 8 to 200 µg/mL, demonstrating greater efficacy compared to Gram-negative bacteria [59,66]. Additionally, certain amino acids and their positions play a crucial role in the antibacterial activity of Mel. For example, the absence of a proline residue at position 14 significantly decreases antimicrobial activity [69]. Further studies have identified nine polymorphisms in the coding region of the Mel gene, including one that results in the substitution of the amino acid serine (Ser) with asparagine (Asp), which could affect the biological activities of Mel. The Ser variant (Mel-S) demonstrated greater cytotoxicity compared to the Asp variant (Mel-N) against *E. coli* [70].

These beneficial effects of BV and its primary compound, Mel, can be utilized as treatments for both humans and animals.

For example, a study showed that both BV and Mel are effective antimicrobial agents against *B. burgdorferi*, the bacterium that causes Lyme disease, a multisystemic illness transmitted by ticks.

The antimicrobial activity of isolated Mel and BV was compared with that of individual and combined antibiotics, such as doxycycline, cefoperazone, and daptomycin. The results showed that both BV and Mel had significant effects on all forms of *B. burgdorferi,* whereas control antibiotics, whether used alone or in combination, demonstrated limited efficacy against biofilm formation [71].

Additional studies have highlighted the antibacterial activity of raw BV and its two major biopeptides: Mel and PLA2. These compounds were tested individually against oral pathogens responsible for dental caries, including *Lactobacillus casei*, *Streptococcus salivarius*, *S. mutans*, *Staphylococcus mitis*, *Staphylococcus sobrinus*, *Staphylococcus sanguinis*, and *Enterococcus faecalis*. Mel was found to be twice as active as raw BV against the tested bacteria (from 4 to 40 µg/mL). PLA2, however, was only active against *L. casei* at concentrations >400 µg/mL [72,73]. Similarly, Mel showed antimicrobial activity against *Staphylococcus* strains and MRSA, while PLA2 exhibited no effect on these strains [70].

In another study, nanoliposomes loaded with BV (BV@NLs) were developed, and their anticancer activity was evaluated in vitro against the HepG-2, MCF-7, and HCT-116 cell lines. BV@NLs displayed spherical shapes with an average size of 230.9 ± 5.21 nm and a positive ζ potential of 46.5 mV.

Compared to raw BV, BV@NLs showed enhanced anticancer activity, with selective cytotoxicity particularly effective against HCT-116 cells (IC50 of 4.16 μg/mL). Furthermore, BV@NLs modulated the expression of apoptosis-related genes. Nanoliposomal encapsulation significantly enhanced the in vitro anticancer activity of BV against the tested cell lines [74].

A further study examined the use of acupuncture with BV for treating eczema on the hands in two clinical cases. The first case involved a 56-year-old woman with symptoms such as itching and erythema, while the second case involved a 33-year-old man with itching and scaling on both hands. The patients were treated for three months with a combination of BV and the herbal decoction San Wu Huangqin (SWH). The treatment led to the complete resolution of lesions, with no relapses reported during follow-up at 1 and 3 years [75].

Expanding on the topic of skin diseases, a pre-clinical study was conducted in mice, where they were repeatedly administered 2,4-dinitrochlorobenzene (DNCB) to induce contact dermatitis. Co-administration of BV and SWH synergistically improved the clinical symptoms of dermatitis. Additionally, these substances improved histological changes in the skin, suppressed immune cell infiltration, and reduced inflammatory cytokines and serum immunoglobulin E. These last two studies suggest that BV and SWH could be alternative treatments for eczema and contact dermatitis [75].

Among the most common diseases affecting adolescents is acne. Acne is an inflammatory skin disorder involving the hair follicle and the associated sebaceous gland. Antibiotics are one of the most frequently used treatments for this skin condition. However, bacterial resistance can sometimes occur [76]. In a randomized, two-arm clinical study with 12 acne patients, skincare products with or without BV were tested. The results showed a 57.7% decrease in ATP, measured to evaluate the reduction in skin microbes. Thus, cosmetics and skincare products containing BV may be promising therapeutic agents for acne [77].

Finally, it is important to note that these bio-peptides, including Mel, the most toxic component of BV, can be harmful and act as toxins if administered improperly. Bee stings can lead to a variety of clinical manifestations, including local inflammatory reactions, allergic reactions, anaphylactic shock, and systemic toxicity [78].

### Bee Venom: Therapeutic Potential in Veterinary Medicine

The substances extracted from BV can be integrated into animal diets to improve reproductive performance [79] and health benefits [80], including the prevention and treatment of diseases. Additionally, they potentially exhibit antimicrobial activity [81]. BV has been used since ancient times to treat various human and animal pathologies. Particularly in the veterinary field, it has demonstrated numerous applications across different species, especially in dogs, rabbits, mice, chickens, rats, and pigs. In dogs, BV has shown anti-inflammatory and analgesic effects, as well as therapeutic effects on facial paralysis.

A study was conducted on a 6-year-old male neutered Shih Tzu dog with left-side facial paralysis and head tilt. Neurological examination revealed a lack of facial sensation and blink reflex. MRI identified an intracranial intra-arachnoid cyst (IIAC), and the provisional diagnosis, after excluding other causes, was idiopathic facial paralysis. Initial treatment with steroids and diuretics yielded minimal results. However, acupuncture with BV led to gradual improvement. The protocol included two sessions per week for five weeks, followed by one weekly session for three additional weeks. Electroacupuncture was initially used, but the treatment was switched to BV acupuncture due to the dog’s intolerance to electrostimulation. Specific points on the animal were treated by injecting 0.1 mg of BV per session. Improvements were evident after the fifth session, with enhanced skin sensation and voluntary eye closure. The blink reflex was recovered after the sixth session. After eight weeks, the dog’s facial symmetry and sensitivity nearly returned to normal without recurrences or side effects. This clinical case demonstrates that BV acupuncture, combining mechanical and pharmacological stimulation from bioactive compounds in BV, can be an effective therapy for idiopathic facial paralysis in dogs [82].

In male rabbits, BV positively impacted reproductive performance and immune response [83]. A study aimed to investigate the effects of different doses of BV on reproductive performance and immune response during the summer season. Forty-eight male rabbits from the V-line were randomly assigned to four groups of 12. Three groups received subcutaneous BV injections at 0.1 (G1), 0.2 (G2), and 0.3 (G3) mg/rabbit twice a week for 20 weeks. The control group (G0) did not receive BV. The BV-treated groups showed significantly shorter reaction times, indicating increased libido compared to the control. Sperm viability, total sperm production, live sperm, and fertility rates were significantly (*p* ≤ 0.05) higher in the BV groups than in the control group. Additionally, levels of testosterone and other biochemical blood constituents, including total protein, albumin, and glucose, were significantly (*p* ≤ 0.05) elevated in the BV groups. BV doses significantly (*p* ≤ 0.05) increased antioxidant indices (Total antioxidant capacity—TAC, Glutathione S-transferase—GST, and GSH) compared to the control group. Moreover, immunoglobulin A (IgA) and immunoglobulin M (IgM) levels were significantly (*p* ≤ 0.05) higher in the BV groups. These findings indicate that BV positively influences sperm quality, sexual behavior, blood biochemical parameters, antioxidant levels, lipid peroxidation bio-markers, and immune response in male rabbits. BV could be an effective, safe, and healthy alternative to pharmaceuticals and hormones in rabbit farming, enhancing reproductive performance and immune response, especially under temperature stress [83].

In two studies, BV and its main component, Mel, were tested against infections in a murine model of MRSA, a human pathogen responsible for many soft tissue skin infections and potentially fatal infections [84,85].

The first study investigated the use of BV in the treatment of MRSA-induced pneumonia. The cohort was divided into groups based on the different doses of venom administered (0.125, 0.25, and 0.5 mg/kg) or linezolid in the control group. Results showed that high doses of BV improved the survival rate of mice, comparable to the antibiotic group. Additionally, BV reduced lung inflammation, as indicated by the suppression of inflammation-related genes (Saa3, Cxcl9, Orm1) [84].

The second study explored the effectiveness of Mel in murine models of systemic and cutaneous MRSA infections. The results showed that Mel was effective in damaging the bacterial cell wall. However, unlike Mel, whole BV worsened systemic infections. This phenomenon may be attributed to the presence of PLA2, which could trigger adverse inflammatory reactions [85]. These studies indicate that mel exhibits greater safety and specificity, demonstrating direct antimicrobial activity and reduced toxicity.

Two studies evaluated the use of BV in broiler chickens, exploring its effects on performance, antioxidant activity, and immune function.

The first study assessed BV supplementation in drinking water for broilers. The experimental protocol involved dividing the cohort into three groups. The first and second groups were fed drinking water supplemented with 0.5 mg/L and 1 mg/L of BV, respectively, while the third group served as the control, receiving only water. Results showed a significant increase in body weight in the BV-treated groups. The group receiving 1 mg/L exhibited a significantly greater weight gain compared to the 0.5 mg/L group. Additionally, BV supplementation improved feed intake and antioxidant activity in the chickens. However, no significant changes were observed in liver function markers or blood cell counts. These results suggest that BV supplementation can enhance broiler performance, especially under stress conditions [81].

Another study examined the effect of BV administered as a spray on broiler chicks to reduce antibiotic use. BV improved weight gain, particularly in cases of *Salmonella gallinarum* infection. It also stimulated the immune response by increasing antibody production, T-cell activity, interleukin-18 levels, interferon-γ, and serum lysozyme activity. BV enhanced bacterial clearance and improved survival in the chickens, reducing pathological damage compared to the control group. These findings suggest that BV could be a valuable alternative for improving chicken health and reducing antibiotic dependence in the poultry industry by stimulating the nonspecific immune response [86].

BV has also shown therapeutic effects in pigs by promoting antibody production and viral clearance in PRRS virus infections [87]. In particular, one study administered BV encapsulated in chitosan/alginate nanoparticles (CH/AL-BV) intranasally to pigs to improve immune response and clearance of the porcine reproductive and respiratory syndrome virus (PRRSV). The results indicated that when combined with PRRSV vaccination, CH/AL-BV treatment significantly enhanced Th1-related immune responses, including an increase in CD4+ T lymphocytes and higher levels of cytokines such as interferon-gamma (IFN-γ) and interleukin-12 (IL-12). Furthermore, there was a notable increase in PRRSV-specific IgG levels. In PRRSV testing, the CH/AL-BV-treated group showed a significant reduction in viral load in serum and lung tissues, with less severe interstitial pneumonia. High levels of PRRSV-specific IgG and neutralizing antibodies were observed. The treatment stimulated significant immune responses, including activation of CD4+ T cells and secretion of IFN-γ, while reducing the immunosuppressive responses typical of PRRSV, such as regulatory T cells and related cytokines (IL-10 and TGF-β). Intranasal administration of CH/AL-BV enhanced immune responses and PRRSV clearance, suggesting a potential strategy to overcome the limitations of traditional vaccination. Moreover, the experimental treatment demonstrated potential as a preventive agent against PRRSV and other viral infections with immunosuppressive characteristics [87].

Finally, BV has shown significant effects in rats, including gastro-protective effects [88], anti-diabetic properties [89], analgesic effects [90], and antibacterial activity against *Staphylococcus aureus* [91]. A preclinical study demonstrated the hypoglycemic effect of BV in alloxan-induced diabetic male rats. Diabetes is a chronic condition characterized by high blood glucose levels (hyperglycemia) due to altered insulin function or quantity. BV containing Mel and PLA2 stimulates insulin secretion from pancreatic β-cells. That study involved eighteen adult male rats (200 ± 20 g), randomly divided into three groups: a control group; a diabetic group induced by alloxan monohydrate; and a treatment group receiving daily BV before feeding for four months. Forty-eight hours after the last injection, blood samples were taken from their hearts, and serum glucose, insulin, triglyceride, and total cholesterol levels were measured. The treated group showed significantly reduced serum glucose, triglycerides, and total cholesterol levels compared to the diabetic group (*p* < 0.01). Additionally, BV treatment resulted in a significant increase in serum insulin levels compared to the diabetic group (*p* < 0.05). These findings suggest that BV may offer a promising therapeutic option to reduce blood glucose and lipid levels in diabetic rats [89].

Table 2 summarizes the key compounds, antimicrobial properties, and veterinary applications of bee venom (BV).

## 4. Propolis: Therapeutic and Antimicrobial Properties with Applications in Medicine

Propolis is a natural product primarily derived from resins collected from the exudates, buds, and shoots of plants. Bees mix these resins with glandular secretions and wax to produce a material that is subsequently used to seal cracks in the hive, create optimal climatic conditions, and protect the colony from potential invaders. It can be considered a social immunity agent [92].

The chemical composition of propolis, like that of honey and all hive products, is influenced by various factors such as geographic, climatic, and botanical variables [93]. It primarily consists of resins (42–58%), wax (33–47%), pollen (3–5%), and a small percentage of additional organic compounds, including ketones, lactones, steroids, and sugars (2–5%) [94,95].

Among the compounds in propolis of major interest are flavonoids, phenolic acids, and terpenoids, known for their antimicrobial, antioxidant, and anti-inflammatory properties. Key flavonoids include galangin, pinocembrin, and chrysin, which are responsible for biological activities [96,97,98]. Numerous studies have reported the antibacterial activity of pinocembrin against various bacterial strains such as *S. mutans*, *S. sobrinus*, *S. aureus*, *E. faecalis*, *L. monocytogenes*, *P. aeruginosa*, and *K. pneumoniae* [99,100]. Phenolic acids, including caffeic acid and its phenethyl ester (CAPE), play a crucial role in antimicrobial activity and antioxidant capacity [25,96,97,98,99,100].

From an antimicrobial perspective, propolis exhibits broad-spectrum activity against a wide range of microorganisms. Extracts, particularly those obtained with solvents such as ethanol, are effective against Gram-positive bacteria such as *Staphylococcus aureus* and *Bacillus subtilis*. However, Gram-negative bacteria are generally more resistant, likely due to their outer membrane, rich in lipopolysaccharides [101]. Antifungal effects of propolis have been observed against various *Candida* strains, including *Candida albicans*, with mechanisms involving damage to the cell membrane and inhibition of ergosterol synthesis [101,102].

The mechanism of action of propolis against microorganisms has been extensively studied and demonstrated. Its components act by increasing cell membrane permeability, altering membrane potential, and inhibiting ATP production [1,9]. Furthermore, some flavonoids interact with microbial DNA, causing toxic effects that lead to cell death [103,104]. Therefore, propolis exhibits various antibacterial mechanisms, including inhibition of cell division, collapse of microbial cell membranes and walls, reduction in bacterial motility, enzymatic inactivation, bacteriolysis, and inhibition of protein synthesis [105].

In vitro studies have highlighted the potential synergistic effect of propolis with antibiotics. Combinations of propolis extracts with drugs such as gentamicin and vancomycin have shown enhanced antimicrobial activity against resistant strains, including methicillin-resistant *Staphylococcus aureus* (MRSA) [106]. This makes propolis a promising agent not only for standalone treatments but also as an adjunct in antibiotic therapy.

In addition to its antimicrobial properties, propolis possesses strong antioxidant power, primarily derived from flavonoids and phenolic acids. These compounds are capable of neutralizing free radicals, thereby protecting cells from oxidative damage. This antioxidant capacity, combined with its antimicrobial properties, opens up applications for propolis in the medical, cosmetic, and food industries.

Recent studies have also shown that propolis can be used as a natural preservative due to its ability to inhibit microbial growth in food. This further consolidates its position as an ecological and safe alternative to chemical preservatives [107,108].

In summary, propolis is a highly complex natural product with significant potential. Its antimicrobial properties, attributed to its multitude of chemical components, make it a promising tool for both medicine and the food industry, with substantial scope for further research and applications. Its medical application has primarily focused on the orthodontic field.

A double-blind, randomized clinical trial aimed to test the effectiveness of a toothpaste containing Brazilian red propolis in preventing dental plaque formation and *Lactobacillus* spp. infection in patients with fixed orthodontic appliances who already had gingivitis and visible plaque at the time of screening. The cohort was divided into two groups: Group 1 used a fluoride toothpaste containing propolis, while Group 2 used a standard fluoride toothpaste. The treatment with propolis-enriched toothpaste for 28 days significantly reduced dental plaque formation and the presence of *Lactobacillus* spp. (*p* < 0.05), whereas the control group showed a significant increase in colony counts (*p* < 0.05) [109]. Similarly, the same toothpaste, in another study, demonstrated effectiveness in orthodontic patients with gingivitis in reducing gingival bleeding and the incidence of infection by *S. mutans* [110]. Therefore, the incorporation of propolis into toothpaste has provided effective antimicrobial activity. In the orthodontic field, the use of propolis may have numerous future applications.

In another study, propolis encapsulated in a nanoparticulate structure was tested to improve the non-surgical treatment of periodontal pockets following standard treatment, which consisted of tartar removal. Patients enrolled who had at least one periodontal pocket were randomly assigned to two different groups.

The first group received the experimental treatment, while the second, representing the control group, was treated with a saline solution. Patients were evaluated at baseline, one month, and three months post-treatment. The results indicated a significant improvement in clinical parameters (*p* < 0.05) in the experimental sites compared to control sites. The gingival index at one month and three months was significantly better in the experimental group. The sub-gingival administration of propolis nanoparticles showed promising results when added to tartar removal in patients with periodontitis who had periodontal pockets [111].

Sy et al. in 2006 and Farias et al. in 2014 explored the anti-inflammatory activity of propolis and subsequently tested its efficacy in a murine model of allergic asthma [112,113]. The results of these experiments were so promising that they encouraged the scientific community to explore the use of propolis in pulmonary diseases affecting humans.

A randomized clinical trial assessed the efficacy of propolis in moderate persistent asthma. Fifty-five participating patients were randomly divided into two groups: the first group received 75 mg of propolis three times a day, while the second group received a placebo, serving as the control group. Significant increases in FEV1, the FEV1/FVC ratio, and expiratory flows were observed, while levels of fractional exhaled nitric oxide (FENO) decreased in the experimental group compared to the control group. Additionally, eosinophilia in sputum in the group that received propolis decreased significantly, in contrast to the control group, which experienced worsening [114].

Continuing in the field of respiratory diseases, propolis has also been tested in ventilator-associated pneumonia (VAP). VAP represents a severe complication associated with mechanical ventilation. Factors such as the insertion of the endotracheal tube and the duration of mechanical ventilation increase the risk of developing VAP. The transmission of VAP occurs through the aspiration of colonized microorganisms residing in the oropharynx, as well as in the stomach and intestines [115]. The use of chlorhexidine mouthwash or gel is widely used to reduce the incidence of respiratory infections. In this study, the anti-inflammatory and antimicrobial effects of chlorhexidine were compared with those of propolis.

Nayereh Darbanian et al. examined 110 patients, half of whom used propolis, and the other half used chlorhexidine as a mouthwash. The incidence of VAP in the intervention group compared to the control group was 10.9% versus 30.9% on the third day (*p* = 0.0166), 23.6% versus 43.6% on the fifth day (*p* = 0.0325), and 25.5% versus 47.3% on the seventh day (*p* = 0.0224). In conclusion, propolis mouthwash may be considered an alternative to chlorhexidine mouthwash for intensive care unit patients [116].

Propolis has also been tested as a potential natural antiseptic. In a double-blind, randomized clinical trial, propolis was used to prevent catheter exit-site infections and peritonitis in patients undergoing peritoneal dialysis. The 90 enrolled patients were divided into three groups: G1 (placebo, normal saline solution), G2 (control, mupirocin), and G3 (experimental, propolis-based ointment). The treatment involved washing the catheter exit site with saline solution and applying one of the three treatments every two days for six months. The results showed that no patient in the propolis group developed catheter exit-site infections or peritonitis, whereas such complications occurred in 10% and 6.7% of patients in the placebo group and 6.7% in the control group. However, the differences between the groups were not statistically significant. Despite these results, this study highlights the advantages of propolis over mupirocin, being natural, cost-effective, and not associated with the development of antimicrobial resistance. Future studies are recommended to confirm these findings [117].

Recent clinical studies have tested the use of propolis in irritable bowel syndrome (IBS). The results showed a significant reduction in symptoms related to pain compared to those who took the placebo (*p* = 0.015). In conclusion, propolis may represent a promising adjunct therapy for alleviating the symptoms of irritable bowel syndrome, particularly for reducing abdominal pain [118].

Thus, propolis emerges as a natural product that is applicable in numerous medical fields. Recent studies have identified its functionality in managing specific conditions such as gingivitis, allergic asthma, respiratory infections, and irritable bowel syndrome, as well as in preventing infections associated with diabetes mellitus. All these findings allow us to recognize propolis as a natural remedy that can also be used in conjunction with conventional therapies.

### Propolis: Therapeutic Applications in Veterinary Medicine

The use of propolis in animals has proven to be a promising alternative to conventional antimicrobials. For instance, in an in vitro and ex vivo study, the antifungal effect of Brazilian red propolis was investigated against *Paracoccidioides brasiliensis*, the causative agent of paracoccidioidomycosis. The in vitro analysis confirmed the antifungal activity of Brazilian propolis with dose-dependent effects. In mouse splenic cells, the experimental compound induced an increase in mitochondrial activity without any cytotoxic effects. The results suggest that Brazilian red propolis not only protects splenic cells from cytotoxicity but also enhances their metabolic activity, improving their ability to combat *P. brasiliensis*. This combined effect indicates that it may function as an immunomodulatory agent, boosting the host’s response to the fungus [119].

Propolis has also demonstrated antifungal activity against vulvovaginal candidiasis caused by *Candida albicans* in a murine model. Amanda Pohlmann Bonfim et al., after confirming the in vitro activity of propolis against *C. albicans* strains, including those resistant to fluconazole, proceeded to test propolis in mice. The infection was induced by vaginal inoculation of *C. albicans*, and the animals were treated with various formulations: pure propolis; a thermoresponsive mucoadhesive system containing propolis (MTS-PRP) at 14%, MTS-PRP at 16%; and nystatin as a positive control. After 7 and 14 days, analyses revealed that fungal load was reduced in all experimental groups, particularly in the MTS-PRP 16% group, which led to an improvement in vaginal epithelial structure comparable to nystatin treatment [120]. The same antifungal activity was confirmed in another study where a mucoadhesive gel formulation containing propolis was tested [121].

Additionally, the antifungal activity of a specific component of propolis, caffeic acid phenethyl ester (CAPE), was tested against oral candidiasis caused by *Candida albicans*. CAPE is one of the bioactive compounds in propolis with various properties, including antiviral, antifungal, immunomodulatory, antioxidant, and anti-inflammatory activities [122,123,124,125]. In oral candidiasis murine models, the application of CAPE significantly reduced the presence of *C. albicans* in the oropharynx, improving typical signs of infection, including decreased inflammatory infiltrates in the tissues. CAPE also demonstrated an immunomodulatory effect, as indicated by the expression of β-defensin 3 in infected mice, a protein involved in the immune system [126]. In rat models, CAPE was also tested in combination with the antifungal amphotericin B, showing renal protective effects against potential toxicity caused by the antifungal [127]. Propolis and its bioactive component CAPE have exhibited antimicrobial properties capable of enhancing immune responses in the animals studied. Future developments could focus on combining propolis and its components with other antifungal drugs to highlight the potential benefits of combination therapy and combat bacterial resistance.

The flavonoid-rich components of propolis and other polyphenolic compounds play a key role in wound healing. A randomized preclinical study conducted on male rats demonstrated that the application of a polyvinyl alcohol (PVA) hydrogel containing propolis could serve as a valid alternative to conventional treatments. The results from the burn wound healing model revealed significant wound contraction (*p* < 0.0001) in the group treated with propolis-based gel, characterized by rapid re-epithelialization, comparable to that achieved with a 5% iodine ointment, which served as the control in this study [128]. In a study conducted on 28 sheep, propolis not only promoted wound healing but also showed possible antibacterial activity against *Corynebacterium pseudotuberculosis*. This study was carried out on a farm in Monte Santo, Bahia, Brazil, with 28 mixed-breed sheep of both sexes exhibiting clinical signs of superficial caseous lymphadenitis (CL). The sheep were randomly divided into two groups: the control group (n = 15) received 10% iodine tincture post-surgery, while the experimental group (n = 13) was treated with a green propolis ointment. The results indicated that the post-surgical treatment with green propolis ointment was more effective than the 10% iodine treatment in healing caseous lymphadenitis in sheep. The animals treated with propolis showed faster healing, with a one-week reduction in recovery time compared to those treated with iodine. Additionally, the sheep treated with propolis had faster hair regrowth [129].

Finally, recent studies on the application of propolis in regenerative endodontics have demonstrated that it may represent an effective option for disinfecting root canals, comparable to triple antibiotic paste (TAP). In dog models, propolis promoted a progressive increase in root length and dentin thickness, along with a reduction in apical diameter, with results similar to those obtained with mineral trioxide aggregate (MTA). These findings suggest that propolis could have future applications in the regeneration of immature non-vital permanent teeth, further expanding its potential as a therapeutic bio-material [130].

Table 3 summarizes the key compounds, antimicrobial properties, and veterinary applications of propolis.

## 5. Bee Pollen’s Antibacterial Activity: Bioactive Components and Potential as a Natural Antibiotic Alternative

The pollen present on flowers is collected by field bees and agglutinated by enzymes secreted from the salivary glands and nectar. It is then stored in the corbiculae located on the tibiae of the hind legs of bees to form pollen loads, commonly referred to as “bee pollen”, in the form of granules [131]. Pollen serves as the raw material used by bees to produce bee bread, which is the main food source for the hive [132]. The transformation of pollen into bee bread occurs because of the action of various enzymes and microorganisms, such as *Pseudomonas*, *Lactobacillus*, and *Saccharomyces*, naturally present in the pollen under specific conditions of temperature and humidity (35–36 °C) [133].

BP contains approximately 250 substances, including amino acids, lipids, vitamins, macronutrients, micronutrients, flavonoids, and organic carotenoid pigments, which make it an ideal food source [134]. Its composition varies depending on geographic origin, ecological habitat, season, weather conditions during collection, bee species, and even beekeeping management practices [135].

For centuries, BP has been used for its medicinal and nutritional properties. The ancient Egyptians described it as “a life-giving powder.” In ancient Greece, the pollen granules transported by the bees’ legs were considered to be made of wax. Aristotle, in his *Historia animalium*, noted their resemblance to wax in terms of hardness. Many of the “Fathers of Western Medicine”, such as Hippocrates, Pliny the Elder, and Pythagoras, placed great trust in the healing qualities of BP, often prescribing it to their patients. The large-scale use of bee-collected pollen for human consumption began only after World War II when pollen traps were developed and became widely available [136].

Water, ethanol, and methanol are the most used solvents for extracting BP. Studies have shown that the highest levels of bioactive compounds are found in ethanol or water extracts compared to natural BP [137].

BP has been the subject of several studies evaluating its antibacterial activity against various bacterial strains, showing promising results:

- General Effects: Moroccan BP has demonstrated inhibitory effects against Gram-positive bacteria, such as *Staphylococcus aureus* and *Streptococcus* spp., and Gram-negative bacteria, such as *E. coli* and *Pseudomonas aeruginosa*, with greater efficacy observed against Gram-positive bacteria [136]. Similar results were obtained with Portuguese BP, which showed activity against *Bacillus cereus*, *S. aureus*, *Salmonella typhi*, and *E. coli* [138];
- Influence of Concentration: Another study tested BP extracts at concentrations ranging from 0.02% to 2.5% against various bacteria, including *B. cereus*, *B. subtilis*, *E. coli*, *Salmonella typhimurium*, *S. aureus*, *Yersinia enterocolitica*, *Enterococcus faecalis*, and *Listeria monocytogenes*, finding that antibacterial efficacy was concentration-dependent [139];
- Activity Against Clostridia: BP has also shown antibacterial activity against various *Clostridium* strains, particularly *C. butyricum* and *C. perfringens*, with the latter being the most sensitive [140];
- Variability Based on Origin: Chilean BP extract demonstrated particularly strong activity against *E. coli*, *P. aeruginosa*, *S. aureus*, and *S. pyogenes* [141]. Slovak BP extracts, both ethanolic and methanolic, were more effective against *S. aureus* and *S. enterica*, with the ethanolic extract inhibiting the growth of *L. monocytogenes* but not *E. coli* or *Salmonella enteritidis* [142];
- Role of the Solvent: The solvent used appears to influence antibacterial activity. Ethanol extracts showed greater efficacy against Gram-negative bacteria (e.g., *E. coli*) compared to Gram-positive bacteria (such as *L. monocytogenes*). An ethanol extract of Slovenian BP exhibited higher antibacterial activity against Gram-negative bacteria (*E. coli* and *Campylobacter jejuni*) compared to Gram-positive bacteria (*L. monocytogenes*) [143]. A similar result was observed for BP collected from a region of Chile, which showed antibacterial activity only against *S. pyogenes* and did not exhibit activity against *E. coli*, *S. aureus*, or *P. aeruginosa*. Furthermore, *E. coli* was the most sensitive strain to the 70% ethanol BP extract but was resistant to the 96% ethanol extract, confirming that bacterial strains are solvent- and concentration-specific [144];
- Mechanism of Action: The antibacterial mechanism of BP is not yet fully understood, but it is believed to be associated with glucose oxidase, an enzyme produced by bees found in pollen [145]. Additionally, the activity may be linked to the phenolic content of pollen, particularly phenolic acids and flavonoids, which may act on bacterial cells by disrupting the cytoplasmic membrane, causing the loss of potassium ions and initiating cell autolysis [139]. Some studies suggest that pollen with lower phenolic concentrations may still be highly effective against microorganisms [146,147];
- Bioactive Compounds: BP contains various bio-active compounds, including fatty acids and microbial metabolites, known for their antibacterial activity. These compounds likely contribute to the efficacy of pollen against numerous pathogenic bacteria [148];
- Selectivity of Pollen: Despite its inhibitory effect against many pathogenic bacteria, BP does not appear to act against probiotic bacteria, such as those used in lactic acid starter cultures, suggesting that pollen could be an interesting alternative to antibiotics, capable of combating pathogens without negatively affecting beneficial bacteria [148].

In summary, BP demonstrates considerable antibacterial activity against a wide range of pathogenic bacteria, with its efficacy depending on concentration, origin, and solvent type used. However, further studies are needed to better understand its mechanism of action and optimize its use as a natural alternative to antibiotics.

### Bee Pollen: Therapeutic Potential for Animal Health and Veterinary Applications

In recent years, to counteract the phenomenon of antibiotic resistance, efforts have been made to promote the use of BP and other bee products to enhance performance and strengthen the immune system of animals. BP can be employed for this purpose [27].

A pre-clinical study conducted on broiler chickens highlighted that the integration of BP at various concentrations can lead to several benefits related to animal growth and health. Specifically, the addition of BP to the diet of the experimental group resulted in increased body weight, accompanied by elevated concentrations of hemoglobin, red blood cells, white blood cells, heterophils, and lymphocytes compared to the control group. In contrast, serum levels of uric acid, creatinine, triglycerides, cholesterol, GOT, and GPT in chicks fed with BP were found to be lower than in the control group. This positive effect was attributed to the ability of BP to stimulate the proliferation and differentiation of immune cells [149].

Studies on broiler chickens and other avian species have shown that dietary supplementation with BP can enhance immune response and lymphocyte proliferation and reduce signs of stress [150], as indicated by the heterophils: lymphocytes ratio (H: L), which was significantly lower in the groups fed BP [151]. These benefits stem from the numerous nutrients present in pollen, including flavonoids such as kaempferol, myricetin, luteolin, and quercetin, which are known to improve immune response and prevent allergic reactions [149]. Additionally, BP has positive effects on lipid and antioxidant profiles, improving heat tolerance and reducing lipid peroxidation due to its polyunsaturated fatty acid (PUFA) content [27].

The use of BP in diet also led to increased levels of red blood cells, hemoglobin, and blood proteins, indicating an overall improvement in hematological and immune functions. BP has been documented to stimulate a stronger antibody response, as evidenced by an increase in serum antibody titers, suggesting that BP promotes humoral immunity [149].

Regarding its numerous beneficial activities, BP, together with propolis, has proven effective in treating neuro-inflammation and intestinal dysbiosis induced by propionic acid. Twenty-four male autistic hamsters were divided into four groups of six animals each: the first group, representing the control, was not treated; the second group was treated with propionic acid to induce dysbiosis and neuro-inflammation; the third group received propionic acid and then BP; the fourth group received propionic acid and then propolis. The results indicated that feeding with propolis and RJ effectively reduced signs of neuro-inflammation, as recorded by a decrease in inflammatory cytokines (IL-6, IFN-γ) and an increase in anti-inflammatory cytokines (IL-10). During this experiment, the presence of *Clostridium difficile* was detected in the fecal analysis of all experimental groups except those that consumed BP. This result suggests that BP may have a particularly effective protective effect in modulating the gut microbiota and preventing the proliferation of pathogenic bacteria. However, further studies are needed to confirm the efficacy of BP in various conditions and species, with the aim of optimizing dosages and usage protocols [152].

Moreover, BP has positive effects on various aspects of animal health and performance, including fertility, hormonal balance, liver and kidney health, lipid profiles, and intestinal morphology, with positive impacts also on growth and development. Concerning reproductive performance, the integration of BP at 0.2 g/kg into the diets significantly improved fertility (86.9%) compared to the control group (69.5%). Additionally, the group fed BP and propolis had larger litters, with heavier pups and a higher survival rate at birth (*p* < 0.01). This improvement is attributed to the presence of micronutrients, phytosterols (such as flavonoids and carotenoids), and polyunsaturated fatty acids in BP, which contribute to hormonal balance, particularly estrogen, and improve egg resistance during incubation [153].

From a hormonal perspective, BP is recognized for its positive impact on hormonal metabolism in both animals and humans. In rabbits fed BP (200 mg/kg), higher serum levels of estradiol-17β and lower levels of progesterone were observed compared to the control group. These beneficial effects are attributed to the balanced combination of nutrients, phospholipids, minerals, and antioxidants in BP, which play a key role in the reproductive process and the maintenance of healthy steroid hormone levels [154,155].

Regarding blood proteins and lipid profiles, BP supplementation in rabbit diets resulted in a significant increase in total protein (TP), albumin (Alb), and globulin (Glo) serum levels (*p* < 0.01), with a higher Glo: Alb ratio compared to the control group. Furthermore, BP reduced plasma cholesterol and triglyceride levels due to its polyunsaturated fatty acid and phospholipid content, which counteract lipid peroxidation. Studies on chickens and other animals confirm that BP can lower cholesterol and triglyceride levels, with positive effects on cardiovascular health [156].

The effects on liver and kidney functions are equally significant: BP has been shown to reduce liver damage by lowering Alanine aminotransferase (ALT) and aspartate aminotransferase (AST) enzyme levels in rabbits and chickens (*p* < 0.01). This protective effect may be attributed to the presence of antioxidant flavonoids in BP. Simultaneously, an improvement in kidney functions was observed, with reduced levels of creatinine and uric acid, suggesting enhanced renal filtration rate and protection against nephrotoxicity [157].

Finally, regarding its effects on intestinal morphology, BP supplementation improved the structure of the small intestine, increasing the length and thickness of the intestinal villi. This promoted greater nutrient absorption efficiency, evidenced by an increase in the villus length to crypt depth ratio (VL: CD), indicating better digestive and absorption capacity. Feeding BP thus stimulates intestinal growth and development, enhancing digestive efficiency and intestinal health in chickens [158].

In conclusion, BP represents a promising natural product with considerable potential in both medical and veterinary fields. Studies suggest that the integration of BP into animal diets, at appropriate dosages, can significantly improve the reproductive and immune status of animals, making it a valuable nutritional strategy in animal production.

Table 4 summarizes the key compounds, antimicrobial properties, and veterinary applications of bee pollen (BP).

## 6. Royal Remedy: The Antibacterial Power of Royal Jelly

RJ is an acidic colloid with a white–yellowish color, produced by worker bees through secretion from the hypopharyngeal and mandibular glands [159,160]. It serves as an exclusive food source for the queen bee and is also fed to larvae during the early stages of development as an essential nutrient for their maturation [161,162]. Its chemical composition varies depending on the geographical origin, time of harvest, and collection method. Despite potential differences, RJ generally consists of 60–70% water, 9–18% proteins, 7–18% sugars, and 3–8% lipids [163,164]. It also contains minor components such as minerals, amino acids (eight of which are essential: Val; Leu; Ile; Thr; Met; Phe; Lys; and Trp), various vitamins, particularly from the B-complex group, hormones, enzymes, polyphenols, and minor heterocyclic compounds [165,166,167]. Among the main bioactive constituents of RJ, proteins play an important role due to their antibacterial properties. The glycoprotein family MRJPs forms 82–90% of the protein content in RJ, with nine identified members (MRJP1-MRJP9) [168]. These proteins exhibit various properties, with some promoting cell proliferation and others proving effective in counteracting bisphenol A-induced proliferation in human breast cancer cell lines [169,170,171].

In a pre-clinical study on mice, MRJP1 and MRJP2 were shown to play a crucial role in macrophage activation, promoting the release of tumor necrosis factor-alpha (TNF-α) [172]. Meanwhile, MRJP3 contributes to the regulation of the immune response by suppressing the production of cytokines such as interleukin (IL)-4, IL-2, and interferon-γ (IFN-γ) in T lymphocytes, highlighting its potential as an immunomodulatory agent [173,174]. However, despite promising pre-clinical findings, further studies are required to fully understand the molecular mechanisms involved and to validate the efficacy of these proteins in humans. Future research could open new avenues for the clinical use of MRJPs, such as in the development of immunomodulatory or anticancer treatments.

What is certain, however, is the indirect antibacterial property of these proteins, especially MRJP1. Upon cleavage of MRJP1, four slightly different peptide classes are formed, distinguished as Jelleine-I, Jelleine-II, Jelleine-III, and Jelleine-IV [19,175]. A study tested the antibacterial properties of Jelleines against various Gram-positive (*S. aureus*, *S. saprophyticus*, *B. subtilis*) and Gram-negative (*E. coli*, *Enterobacter cloacae*, *K. pneumoniae*, *P. aeruginosa*) bacteria. The results revealed that Jelleine-I and Jelleine-II exhibited broad-spectrum activity, while Jelleine-III was less active, and Jelleine-IV showed no antimicrobial activity [175]. The antibacterial activity of MRJP1 is attributed to the first three classes of Jelleines.

Royalisin, another antimicrobial protein, works in conjunction with Jelleines to provide broad-spectrum protection [19,176]. Royalisin is an amphipathic peptide composed of 51 amino acids, six of which form three disulfide bonds [177,178]. A study explored the antibacterial efficacy of royalisin and its variant, royalisin-D, against Gram-positive and Gram-negative bacterial strains, including *S. intermedius B* and *P. aeruginosa.* The results showed that both proteins significantly reduced bacterial surface hydrophobicity, with royalisin being more effective than royalisin-D. Furthermore, increased cell membrane permeability was observed, measured by UV absorbance at 260 nm. Both proteins demonstrated strong antibacterial activity against Gram-positive bacteria, while their effectiveness against Gram-negative bacteria was limited. The superior bioactivity of royalisin compared to royalisin-D was attributed to the presence of an intramolecular disulfide bond [178].

Another bio-active molecule with antimicrobial properties in RJ is the fatty acid trans-10-hydroxy-2-decenoic acid (10-HDA), the most abundant component in the lipid fraction [179]. The 10-HDA has shown bacteriostatic activity against both Gram-negative (*E. coli*) and Gram-positive (*M. pyogenes*, *B. subtilis*, *S. aureus*) bacteria [180]. Subsequent comparative studies between ether-soluble fractions rich in 10-HDA and insoluble fractions primarily containing royalisin demonstrated that only the former exhibited significant antibacterial activity, inhibiting the growth of bacteria such as *Streptomyces* and *S. aureus* at concentrations of 30 mg/mL [181]. Furthermore, 10-HDA was found to reduce cellular adhesion and gene expression of *S. mutans*, an oral pathogen, by inhibiting key genes like gtfB and gtfC. Other fatty acids present in RJ, such as 3-hydroxydecane-dioic acid and 10-acetoxy-2-decenoic acid, have shown efficacy against oral pathogens (*S. mutans*, *S. viridans*) and Gram-positive bacteria (*S. aureus*, *S. epidermidis*), but are less active against Gram-negative bacteria, with limited action against *K. pneumoniae* [182].

Consequently, some MRJPs, Jelleines, royalisin, and 10-HDA may act synergistically to provide RJ with effective and promising antibacterial activity. RJ has shown significant antibacterial properties against bacteria associated with periodontal infections, such as *Porphyromonas gingivalis*, *Prevotella intermedia*, *Fusobacterium nucleatum*, and *Aggregatibacter actinomycetemcomitans* [183]. In vitro studies comparing the antimicrobial properties of RJ with chlorhexidine mouthwash, the gold standard for periodontal disease treatment, confirmed its antimicrobial properties. Sub-gingival plaque samples from 15 patients with chronic periodontitis were analyzed to assess the antimicrobial efficacy of RJ and chlorhexidine against aerobic and anaerobic bacteria. The results showed that while chlorhexidine was more effective at lower doses against bacterial activity, RJ exhibited significant activity against anaerobic bacteria. In conclusion, Koshla et al. propose that RJ represents a viable natural alternative for the non-surgical management of chronic periodontitis, suggesting prospects for combined RJ and chlorhexidine treatments [184,185].

In addition to its antimicrobial and anti-inflammatory activities, which have been highlighted in numerous studies [186,187], a study on a group of dogs demonstrated that the water-soluble fraction of RJ contained a cholinergic substance capable of inducing vasodilation in the animal’s femoral artery [188]. Based on these results, Mansour Siavash et al. sought to replicate these effects in the clinical context of diabetic foot ulcers, analyzing 64 cases of ulcers. Their study aimed to test the efficacy of a 5% sterile RJ solution compared to a placebo, evaluating the clinical progression of the lesions. Although the results indicated promising effects of the experimental treatment, no statistically significant superiority was found for the 5% topical RJ compared to the placebo in the treatment of diabetic foot ulcers [189].

Future implications for RJ were identified in a study in which beehive products, in solution with silver nitrate, were used to obtain silver-enriched nanoparticles. This approach was employed to assess the antimicrobial properties of AgNPs against *Candida guilliermondii* NP-4 yeast. The results showed that RJ-mediated AgNPs effectively suppressed yeast growth through mechanisms involving oxidative stress, inhibition of ATPases, and lipid peroxidation [190].

### Exploring the Benefits of Royal Jelly in Veterinary Medicine for Animal Welfare

There are limited data available regarding the application of RJ in veterinary medicine. However, it has been shown to exhibit activity against various types of bacteria, both Gram-positive and Gram-negative. The antimicrobial effect of RJ against MRSA was first analyzed in vitro and then subsequently in vivo. In vitro tests demonstrated that RJ had antibacterial activity, significantly reducing biofilm formation and impacting bacterial adhesion to cells. These anti-biofilm and anti-adhesive effects of RJ were later confirmed in in vivo experiments conducted on rats. RJ, applied at varying concentrations to infected wounds, resulted in a significant reduction in lesion diameter. Furthermore, the treated wounds showed no signs of pus or exudate after the experimental treatment. This study confirmed that RJ not only promoted wound healing but also contributed to the elimination of MRSA-induced infection [191]. Additional in vivo studies have highlighted the antibacterial activity of RJ against *Pseudomonas aeruginosa* and *Listeria monocytogenes*, particularly showing that the bioactive component of type 1 Jelleines played a crucial role in counteracting *Listeria monocytogenes* activity [192,193].

The wound-healing potential of RJ in vivo was further validated by a study conducted by Yan Li et al. [194]. This study group examined RJ derived from the flowers of *Castanea mollissima Bl.* (Chinese chestnut) and *Brassica napus* L. (rapeseed) and assessed its healing potential when applied to rat wounds. The results showed that the application of *Castanea mollissima Bl.* RJ improved wound healing activity, recording better outcomes compared to the control on days 2 and 4 of treatment. In contrast, RJ from *Brassica napus* flowers did not improve wound repair. These results reinforce the theory that the bioactive compounds in all beehive products depend on various factors [194].

Numerous studies have suggested a specific role for RJ in regulating the immune response. It has been reported that RJ affects various aspects of the immune response, modulating histamine production, a key mediator in inflammatory processes, and regulating the levels of inflammatory cytokines, which play an important role in immune cell communication. Furthermore, RJ appears to influence adaptive immune responses by interacting with T-cell proliferation and modulating the synthesis of IgG and IgE antibodies [174,195,196]. Based on the existing literature, Malika Guendouz et al. tested the immune properties of RJ in vivo on 100 mice, assessing the systemic allergic response to cow’s milk proteins [197]. The results were promising. RJ significantly reduced the systemic anaphylactic response by lowering systemic histamine levels. Additionally, it protected the intestine by abolishing ion secretion related to the allergy [197].

While the future of RJ in medical applications holds considerable promise, translating promising in vitro and in vivo findings into practical clinical applications remains a challenge. Although in vitro studies offer valuable preliminary insights, further in vivo research is essential to validate and expand upon these findings. A deeper understanding of RJ’s mechanisms of action through in vivo models is crucial for defining its potential for widespread clinical use.

Table 5 summarizes the key compounds, antimicrobial properties, and veterinary applications of royal jelly (RJ).

## 7. Apitherapy in Veterinary Medicine: Assessing the Future Therapeutic Potential of Bee Products in Animal Health and Clinical Applications

Table 6 provides a detailed overview of the different hive products and their specific uses in Veterinary Medicine. This section describes at length some of them of particular interest.

Clinical trials have examined the effects of honey across various species, including dogs, horses, cats, cattle, and pigs [13]. In a study on horses, infected hind leg wounds treated with manuka honey gel or pure manuka honey healed more rapidly and effectively than untreated control wounds [15]. Remarkable results in wound healing with honey were observed in both cats and dogs, regardless of whether a commercial product containing medical-grade honey (L-Mesitran^®^) was used [16,198,199] or raw honey was applied directly to gauze and then to the wound [14]. L-Mesitran^®^ demonstrated exceptional efficacy in treating cutaneous injuries. For instance, in a case involving a cat with a fractured distal ulna and complete skin loss over 100% of its leg, the leg fully recovered, including bone healing, with new skin and hair regrowth [13]. When compared to *Hypericum perforatum*, which significantly improved tissue perfusion relative to untreated controls, medical-grade honey showed superior results in cats with full-thickness skin wounds. These results included reduced edema, increased angiogenesis, and higher fibroblast concentrations [200]. This is noteworthy, as *H. perforatum* has long been utilized to promote wound healing by enhancing collagen accumulation [201], stimulating tissue repair and regeneration-related gene expression [202], and supporting epithelialization and granulation [203].

Propolis ethanolic extracts have also shown promising efficacy against the etiological agents of bovine mastitis, including *Staphylococcus aureus* and *Escherichia coli*. This potential has attracted significant interest from veterinarians for treating bovine mastitis caused by antibiotic-resistant microorganisms. Such efficacy was demonstrated in vitro against both standard strains and wild types of *S. aureus* and *E. coli* isolated from mastitic milk [204], in complex media or milk [205], and bovine mammary epithelial cells damaged by these pathogens [206]. However, propolis was found to have a negative impact on mammary tissue viability, as it reduced the viability of bovine mammary gland explants [204]. In vivo studies have highlighted propolis’s antiviral properties, particularly against herpes simplex viruses HSV-1 and HSV-2 [207,208]. Additionally, certain forms of propolis have exhibited antiparasitic properties in animal models. For instance, propolis extracts reduced infection levels of the microsporidian parasite *Nosema ceranae* in the intestines of honey bees following oral administration [209,210,211].

Propolis has demonstrated remarkable efficacy in wound healing across numerous animal species [212]. In dogs, a 30% propolis paste significantly shortened the healing time by improving reepithelization and wound contraction [213]. In pigs, propolis-containing ointments proved effective in treating burn wounds [214,215], promoting healing through broad-spectrum antimicrobial action, enhanced neo-angiogenesis, accelerated epithelialization [215], and inhibition of fibronectin production and degradation in the wound area [214].

When added to broiler feed in low dosages (10 mg/kg), propolis effectively prevented pathological lesions, protecting the liver and blood vessels. These benefits were significantly enhanced at a higher dosage (50 mg/kg) [82]. Bee venom has also shown promising therapeutic potential. In a dog with idiopathic facial paralysis, bee venom acupuncture led to full recovery of sensory and neurological facial functions after eight weeks, with gradual improvement in clinical symptoms [83]. Studies on rabbits have demonstrated that bee venom enhances immune response, reproductive performance, and overall health due to its antioxidant properties [81,216]. Similarly, in broiler chickens, bee venom supplementation improved feed conversion and body weight without causing adverse side effects [86,217]. Moreover, bee venom exhibited antibacterial, antifungal, and antiviral properties and acted as an immunoprophylactic agent in dogs [218], broiler chickens [87], and pigs [85]. The peptide melittin holds promise for treating methicillin-resistant *Staphylococcus aureus* (MRSA) infections in animals [219]. Bee pollen’s potential in animal husbandry has been primarily explored in terms of its effects on slaughter yield, meat quality, and growth performance. Supplementing chicken feed with 400–800 mg/kg of bee pollen or its ethanolic extract improved gut microbiota and enhanced animal growth. However, meat performance and slaughter yield were not significantly affected [220]. Even when bee pollen concentrations were increased tenfold (7.5–20 g/kg feed), broiler growth performance, immune response, and gut microbiota showed substantial improvements, though slaughter yield remained unaffected [221,222,223].

It is important to note that honey bee hive products may contain contaminants, including acaricides used to control *Varroa destructor* and pesticides commonly employed in agriculture [224,225]. Products with high-fat content, such as propolis and wax, as well as hive air, are particularly susceptible to contamination [226,227]. Therefore, using natural sources like plants, algae, or fungi [228,229] or adopting traditional beekeeping practices that minimize stress, pathogen exposure, and chemical treatments is recommended to ensure the health and resilience of honey bee colonies [230,231]. Finally, apitherapy in veterinary medicine should only be applied following an examination by a licensed veterinarian. While allergic reactions are less common in animals than humans, they are still possible with any bee product. Immediate veterinary care is crucial if an allergic reaction, such as envenomation from bee venom, is suspected [232,233]. Although apitherapy is currently permitted only as a supplementary treatment, it holds immense potential for veterinary medicine. Increasing evidence highlights the benefits of apitherapy in enhancing animal health and vitality. Consequently, there is growing interest among veterinary professionals and animal owners in incorporating bee products into animal care. However, they must remain vigilant about potential risks, including the rare but potentially fatal anaphylactic reactions [234]. To optimize the use of bee products in veterinary medicine, further preclinical and clinical research is essential to fully understand their mechanisms of action and determine the best dosages and application methods for various species.

## 8. Materials and Methods

This study conducted an extensive review of the existing literature to explore the antimicrobial and therapeutic properties of hive products. Data were gathered through systematic searches in key databases such as Google Scholar, MEDLINE PubMed, SciELO, and SCOPUS. Data extraction was performed with particular attention to studies that examined the effectiveness of hive products against antimicrobial resistance in both human and veterinary contexts. A comprehensive set of search terms was employed to ensure a thorough exploration of the relevant literature. These search terms included, but were not limited to, “hive products”, “honey effect”, “bee product antimicrobial”, “bee products veterinary”, “apitherapy veterinary”, “antibiotic resistance”, “beehive products veterinary”, and “bee products veterinary”. The search strategy involved the use of operators such as “AND” and “OR” to refine the results and ensure a broad yet targeted inclusion of pertinent studies. To maintain the quality and relevance of the selected studies, only those published in English were considered for inclusion in the review. This approach allowed for the extraction of a diverse and representative range of studies, contributing to a comprehensive understanding of the topic under investigation. Six tables summarizing what is in the text are included.

## 9. Conclusions

The increasing threat of antimicrobial resistance has spurred a renewed investigation into the bioactive potential of natural products, particularly those derived from beehives. Recent studies highlight the therapeutic promise of honey, BV, propolis, BP, and RJ, emphasizing their applications in medicine, veterinary science, and pharmacology. These substances, especially in combination with existing antimicrobials, have shown encouraging results in combating drug-resistant microorganisms, opening new avenues for antimicrobial therapy.

This review has examined the antimicrobial properties and veterinary applications of beehive products. While demonstrating significant potential in mitigating resistance, their efficacy is influenced by compositional variations and preparation methods. The bioactive components within these products are subject to variability based on environmental factors, geographical origin, and processing techniques, posing challenges for standardization in clinical settings.

Future research must address these challenges by further exploring the inherent variability and complexity of beehive products. Standardizing production methods and identifying optimal conditions for maximizing bioactive potential are crucial. This necessitates more rigorous studies to evaluate the consistency of antimicrobial properties across diverse samples and conditions. Furthermore, the limitations of small sample sizes in existing research must be addressed through larger, more standardized clinical trials to ensure robust, reproducible data. Addressing these research gaps will facilitate the integration of beehive products into clinical practice, potentially providing effective natural alternatives for treating microbial infections, especially in the context of rising antimicrobial resistance.

## Figures and Tables

**Table 1:** Antimicrobial Properties and Veterinary Applications of Honey.

Hive Products	Bee Species	Geographical Region	Specific Physicochemical Characteristics	Antimicrobial Properties	Medical Applications	References
Manuka honey	*Apis mellifera*	New Zealand (origin of Manuka)	Contains methylglyoxal; high osmotic potential.	Inhibits growth of *Staphylococcus pseudintermedius* (in vitro)	Topical treatment for infections in humans and animals; potential antibiotic adjuvant for resistant strains	[46]
Manuka honey	*Apis mellifera*	New Zealand (origin of Manuka)	Contains methylglyoxal (MGO 830+)	*Staphylococcus aureus*, *S. epidermidis*, *S. lugdunensis* (in vitro)	Topical applications; inhibits biofilm formation	[47]
Manuka honey	*Apis mellifera*	New Zealand (origin of Manuka	Non-peroxide activity due to phytochemicals and methylglyoxal	*Streptococcus mutans* (in vitro)	Potential use for dental health: reduces biofilm and adherence of cariogenic bacteria	[48]
Commercial MediHoney	Not specified	Germany	High osmolarity, low pH, glucose oxidase activity	*Staphylococcus aureus MRSA* (in human clinical trial)	Wound healing, eradication of MRSA	[50]
Commercial Medical Grade Honey	Not specified	Not specified	High osmolarity, hydrogen peroxide activity, acidic PH	*Candida* spp. (in human clinical trial)	Treatment of vulvovaginal candidiasis	[51]
Medical grade honey (MGH)	Not specified	Commercial MGH (L-Mesitran Soft, Theo Manufacturing B.V., Maastricht, the Netherlands)	Medical-grade honey (strong antimicrobial and healing properties)	*Enterococcus faecalis*, *Aeromonas hydrophile* (in white rhinos)	Treatment of severe wounds (e.g., gunshot and poaching injuries)	[53]
Unheated pure honey	Not specified	Not specified	Unprocessed honey, high sugar content, low pH, hydrogen peroxide production	No bacterial growth was detected (in white New Zealand rabbits)	Topical treatment of cutaneous wounds	[54]
Honey	Not specified	Saudi honey: Alnahal Aljawal honey (Wadi) or Bin Ghaithan honey (Talh)	Honey rich in antioxidants: flavonoids, phenolic acids, tocopherols and ascorbic acid	Preventive antimicrobial activity (in rats)	Protecting against indomethacin-induced gastric	[56]
Medical grade honey (MGH)	*Apis mellifera*	Commercial MGH(Covetrus, Dumfries, Scozia)	MGO interferes with inflamed protein	No bacterial growth was detected in synovial cultures collected before or during surgery (in horses)	Manuka honey applied inside inflamed synovial	[57]

**Table 2:** Antimicrobial Properties and Veterinary Applications of Bee Venom.

Hive Products	Bee Species	Geographical Region	Specific Physicochemical Characteristics	Antimicrobial Properties	Medical Applications	References
Bee venom (commericial melittin)	*Apis mellifera*	Purchased from Sigma-Aldrich (St Louis, MO, USA)	Amphipathic peptide, water-soluble, toxic at high concentrations	*S. aureus*, *E. coli*, *P. aeruginosa,* and *Bacillus subtilis* (in vitro)	Potential for treating bacterial biofilms	[66]
Commercial Bee venom	*Apis mellifera*	Purchased from Sigma-Aldrich (St Louis, MO, USA)	Contains melittin	*Borrelia burgdorferi* (in vitro)	Potential treatment for antibiotic-resistant *Borrelia burgdorferi* infections (e.g., Lyme disease) [71]	[71]
Bee Venom	*Apis mellifera*, *Apis cerana*	Not specified	Melittin is the major active component (40–60% of venom).	*E. Coli* (in vitro)	Potential anti-inflammatory agent (inhibits IL-6 and TNF-α)	[70]
Bee Venom	*Apis mellifera*	South Korea	Composed mainly of melittin and other bioactive peptides (apamin, adolapin).	*Propionibacterium acnes* (in vitro and in human clinical trial)	Topical treatment for acne vulgaris: reduces inflammatory and non-inflammatory lesions	[77]
Bee venom	Not specified	Not specified	Bee venom used in acupuncture (0.1 mg per acupoint, containing melittin and phospholipase A2)	Not applicable (nerve disorder study in dogs)	Treatment of idiopathic facial paralysis	[82]
Bee venom	*Apis mellifera*	China	Bee venom injections (0.1–0.3 mg/rabbit, containing melittin and apamin)	Not applicable (reproductive study in rabbits)	Enhancing reproductive performance and immunity	[83]
Bee venom	*Apis mellifera*	Korea	Purified bee venom (60.2% melittin content)	Methicillin-resistant *Staphylococcus aureus* (MRSA) (in mice)	Treatment of pneumonia due to MRSA infection	[84]
Bee venom	*Apis mellifera*	Korea	Phospholipase A2 and the melittin peptide	Methicillin-resistant *Staphylococcus aureus* (MRSA) (in mice)	Melittin as an antimicrobial agent for MRSA wound healing	[85]
Bee venom	*Apis mellifera*	Not specified	Phospholipase A2 and the melittin peptide	Not applicable (growth study in fast-growing broilers)	Immune system enhancement	[86]
Bee venom	*Apis mellifera*	Not specified	Chitosan/alginate nanoparticle-encapsulated bee venom	Not applicable (viral study in pigs)	Immune system enhancement, increased PRRSV-specific antibodies, and mitigated pneumonia signs	[87]
Bee venom	*Apis mellifera*	Khuzestan, Iran	Venom containing melittin and phospholipase A2	Not applicable (study on glycemic effect in mice)	Honeybee venom (apitoxin) can be used as therapeutic option to lower blood glucose	[89]

**Table 3:** Antimicrobial Properties and Veterinary Applications of Propolis.

Hive Products	Bee Species	Geographical Region	Specific Physicochemical Characteristics	Antimicrobial Properties	Medical Applications	References
Propolis	*Apis mellifera*	South Africa (North West Province)	Contains flavonoids: pinocembrin, galangin, chrysin	*Staphylococcus aureus*, *Listeria monocytogenes*, *andida albicans, C. tropicalis*, *Cryptococcus neoformans* (in vitro)	Potential for treating bacterial and fungal infections	[99]
Propolis	*Apis mellifera*	Nepal	Contains flavonoid aglycones (isoflavonoids, neoflavonoids)	*Helicobacter pylori*, *Staphylococcus aureus* (in vitro)	Potential for treating infections caused by Gram-positive bacteria	[100]
Propolis	*Apis mellifera*	Germany, Ireland, Czech Republic	phenolics, flavonoids (chrysin, pinocembrin, galangin), cinnamic acid, and its esters. Total phenolic content is highest in Czech propolis	*Candida Albicans* (in vitro)	Potential to enhance antibiotic efficacy; treat resistant bacterial and fungal infections and combat biofilms	[101]
Red Propolis	Not specified	Marechal Deodoro, Brazil	High flavonoid and isoflavonoid content; presence of quercetin, vestitol, and neovestitol	*Lactobacillus* spp. (human clinical trial)	Reduction in visible plaque index (VPI) and gingivitis control	[109]
Red Propolis	Not specified	Marechal Deodoro, Brazil	High flavonoid and isoflavonoid content; presence of quercetin, vestitol, and neovestitol	*S. Mutans* (human clinical trial)	Reduction in gingival bleeding and control of biofilm	[110]
Propolis	*Scaptotrigona aff. postica*	Barra do Corda, Maranhão, Brazil	Contains phenolic acid, total phenols, and flavonoids	Not applicable, asthma study (in mice)	Reduction in airway inflammation asthma	[112]
Propolis	Not specified	Mashhad, Iran	Caffeic acid phenethyl ester (CAPE), quercetin, and naringenin	Not applicable, asthma study (human clinical trial)	Improves clinical and physiological findings in moderate asthma	[114]
Red Propolis	*Apis mellifera*	Brazil (Northeast region)	Rich in formononetin, vestitol, neovestitol	*Paracoccidioides brasiliensis* (in mice)	Potential therapeutic agent for systemic fungal infections like *paracoccidioidomycosis*	[119]
Propolis	*Apis mellifera*	Brazil	Mucoadhesive thermoresponsive propolis extract (14–16%)	*Candida albicans* (in mice)	Treatment of vulvovaginal candidiasis	[120]
Green Propolis	Not specified	Bahia State, Brazil	Green propolis contains Artepillin C, flavonoids	*Corynebacterium pseudotuberculosis* (in sheep)	Wound healing	[129]

**Table 4:** Antimicrobial Properties and Veterinary Applications of Bee Pollen.

Hive Products	Bee Species	Geographical Region	Specific Physicochemical Characteristics	Antimicrobial Properties	Medical Applications	References
Bee pollen	Not specified	Not specified (general review, including studies globally)	Rich in polyphenols, flavonoids, vitamins (A, C, E), minerals (K, Zn, Fe), and proteins	*Staphylococcus aureus*, *Streptococcus* spp., *Escherichia coli*, *Pseudomonas aeruginosa* (in vitro)	Antimicrobial activity against various pathogens; antioxidant properties	[136]
Bee pollen	Not specified	Portugal	Phenolic content: 10.5–16.8 mg GAE/g; antioxidant activity	*Escherichia coli*, *Bacillus cereus*, *Salmonella typhi* (in vitro)	Possible source for antimicrobial agents	[138]
Bee pollen	Not specified	Central Slovakia	Polyfloral bee pollen; extracted with ethanol, evaporated under vacuum, and dissolved in DMSO (50%, 25%, 12.5%, 6.25%)	*Clostridia butyricum*, *C. histolyticum*, *C. intestinale*, *C. perfringens*, *C. ramosum* (in vitro)	Potential combined use with antibiotics to combat antibiotic resistance	[140]
Bee pollen	Not specified	Chile, Calera de Tango	Monofloral (60% *Azara petiolaris*); aqueous extract concentration: 82.4 mg/mL	*Staphylococcus aureus* and *Streptococcus pyogene* (in vitro)	Potential for pathogen control and development of natural products	[141]
Bee pollen	Not specified	Kafrelsheikh, Egypt	Bee pollen contains 62.82% carbohydrates, 19.23% proteins, 4.09% lipids, 2.28% ash, and various amino acids	Not applicable (growth study in broiler chickens)	Bee pollen supplementation improved growth performance, immune system efficiency, and carcass traits	[149]
Bee pollen	Not specified	Saudi Arabia	flavonoids, fatty acids, and phytosterols, including quercetin	*Clostridium Difficile* (in mice)	Gastrointestinal health	[152]

**Table 5:** Antimicrobial Properties and Veterinary Applications of Royal Jelly.

Hive Products	Bee Species	Geographical Region	Specific Physicochemical Characteristics	Antimicrobial Properties	Medical Applications	References
Royal Jelly	*Apis mellifera*	Brazil	Four short cationic peptides (Jelleine-I, -II, -III, -IV)	*S. aureus*, *E. coli* and *Candida albicans* (in vitro)	Potential model for developing new antimicrobial agents due to broad-spectrum activity	[19]
Royal Jelly	*Apis mellifera*	Singapore	Proteins, peptides, and 10-hydroxy-2-decenoic acid (10-HDA)	*Porphyromonas* *gingivalis*, *Prevotella intermedia*, *Fusobacterium* *nucleatum* (in vitro)	Potential use in treating periodontal disease as an adjunct to conventional therapy	[183]
Royal Jelly	*Apis mellifera*	Not specified	Royalisin, an antimicrobial peptide (5.5 kDa) rich in cysteine; forms a compact structure stabilized disulfide bonds	*Staphylococcus* *intermedius* B, *Pseudomonas* *aeruginosa* and *Staphylococcus aureus* (in vitro)	Potential for combating bacterial pathogens	[178]
Royal Jelly	*Apis mellifera*	Not specified	Major fatty acid component (10-Hydroxy-Δ2-decenoic acid);	*E. coli, S. aureus*, *Bacillus subtilis* and *Streptococcus piogene* (in vitro)	Antimicrobial potential and supports immune defenses	[180]
Royal Jelly	*Apis mellifera*	Two samples: China and Egypt	Rich in proteins (27–41%), carbohydrates (30%), lipids (8–19%), vitamins, and 10-Hydroxydecanoic acid (HDA)	*Escherichia coli*, *Pseudomonas aeruginosa*, *Staphylococcus aureus*, *Bacillus subtilis*, *Candida albicans*, *Aspergillus fumigates* (in vitro)	Antimicrobial agent, potential application for wound care, antifungal treatments, and immune support	[181]
Royal Jelly	*Apis mellifera*	Not apecified	Isolated 10-Hydroxy-2-decenoic acid (HDA)	*Streptococcus* *mutans* (in vitro)	Potential use in preventing dental caries and biofilm-related oral diseases	[182]
Royal Jelly	Not specified	Kotayk region, Armenia	Contains flavonoids and phenolic compounds	*Candida* *guilliermondii* (in vitro)	Potential antifungal applications, understanding oxidative stress in yeast cells	[190]
Royal Jelly	Not specified	Egypt	Active proteins such as royalisin, 10-hydroxy-2-decenoic acid, and various fatty acids	*Methicillin-resistant Staphylococcus aureus* (in mice)	Biofilm disruption	[191]
Royal Jelly	*Apis mellifera*	South China (Zhejiang)	Water, proteins, carbohydrates, lipids, vitamins, free amino acids, and minerals	No bacterial growth was detected (in rats)	Wound healing	[194]
Royal Jelly	*Apis mellifera*	Arta region, Greece	10-hydroxy-2-decenoic acid (10-HDA) and 3,10-dihydroxy-decanoic acid (3,10-DDA)	Not applicable (study on immune activity in vitro and in rats)	Immune modulation	[195]
Royal Jelly	Not specified	Mitidja Plain, Algeria	Proteins (12–15%), sugars (10–16%), lipids (3–6%), and polyphenolic compounds	Not applicable (study on immune activity in mice)	Preventive effects against systemic and intestinal immune responses in cow’s milk allergy; reduced plasma histamine and specific IgE/IgG levels	[197]

**Table 6:** Use of Hive Products in Veterinary Medicine.

Bee Product	Geographical Region	Physicochemical Characteristics	Bacterial Species	Veterinary Applications	Animal	References
Medical grade honey (MGH)	Commercial MGH (L-Mesitran Soft, Theo Manufacturing B.V., Maastricht, the Netherlands)	Medical-grade honey (strong antimicrobial and healing properties).	*Enterococcus faecalis*, *Aeromonas hydrophile* and various wound-associated bacteria.	Treatment of severe wounds (e.g., gunshots and poaching injuries).	White rhinos	[53]
Unheated pure honey	Not specified	Unprocessed honey, high sugar content, low pH, hydrogen peroxide production.	No bacterial growth was detected.	Topical treatment of cutaneous wounds.	White New Zealand rabbits	[54]
Medical grade honey (MGH)	Commercial MGH, (L-Mesitran Soft, Theo Manufacturing B.V., Maastricht, the Netherlands	Medical-grade honey (strong antimicrobial and healing properties).	Various wound-associated bacteria.	Intralesional treatment of lacerations.	Horses	[55]
Not specified	Saudi honey: Alnahal Aljawal honey (Wadi) or Bin Ghaithan honey (Talh)	Honey rich in antioxidants: flavonoids, phenolic acids, tocopherols, and ascorbic acid.	Preventive antimicrobial activity.	Protecting against indomethacin-induced gastric ulceration in rats.	Rats	[56]
Medical grade honey (MGH	Commercial MGH(Covetrus, Dumfries, Scozia)	MGO interferes with inflamed protein.	No bacterial growth was detected in synovial cultures collected before or during surgery.	Manuka honey applied inside inflamed synovial.	Horses	[57]
Venom	Not specified	Bee venom used in acupuncture (0.1 mg per acupoint, containing melittin and phospholipase A2).	Not applicable (nerve disorder study).	Treatment of idiopathic facial paralysis.	Dogs	[82]
Venom	China	Bee venom injections (0.1–0.3 mg/rabbit, containing melittin and apamin).	Not applicable (reproductive study).	Enhancing reproductive performance and immunity.	Rabbits	[83]
Venom	Korea	Purified bee venom (60.2% melittin content).	Methicillin-resistant *Staphylococcus aureus* (MRSA).	Treatment of pneumonia due to MRSA infection.	Mice	[84]
Venom	Korea	Phospholipase A2 and the melittin peptide.	Methicillin-resistant *Staphylococcus aureus* (MRSA).	Melittin as an antimicrobial agent for MRSA wound healing.	Mice	[85]
Venom	Not specified	Phospholipase A2 and the melittin peptide.	Not applicable (growth study).	Dietary BV, when added in the range from 10 to 500 μg/kg into broiler feed, increased growth performance.	Broilers	[86]
Venom	Not specified	Chitosan/alginate nanoparticle-encapsulated bee venom (slow release, mucoadhesive).	Not applicable (viral study).	Enhanced Th1 immune response, reduced viral load, increased PRRSV-specific antibodies, and mitigated pneumonia signs.	Pigs	[87]
Venom	Khuzestan, Iran	Venom containing melittin and phospholipase A2).	Not applicable (study on glycemic effect).	Honeybee venom (apitoxin) can be used as a therapeutic option to lower blood glucose and lipids.	male rats	[89]
Propolis	Brazil (Northeast region)	Brazilian red propolis (rich in formononetin, vestitol, neovestitol).	*Paracoccidioides brasiliensis* (fungal study).	Potential therapeutic agent for systemic fungal infections like paracoccidioidomycosis.	Mice	[119]
Propolis	Brazil	Mucoadhesive thermoresponsive propolis extract (14–16%).	*Candida albicans* (fungal study).	Treatment of vulvovaginal candidiasis.	Mice	[120]
Pure substances	Pure substances	Caffeic Acid Phenethyl Ester (CAPE) was studied for its antifungal, antibiofilm, and immunomodulatory properties.	*Candida albicans* (fungal study).	Used for treating oral candidiasis and systemic infections.	*Galleria mellonella* model and mice;	[126]
Propolis	India (Bharatpur, Rajasthan)	Propolis extract containing polyphenols (34.82 mg/g gallic acid equivalent) and flavonoids (23.61 mg/g quercetin equivalent).	No bacterial growth was detected.	Hydrogel for wound healing in burn, excision, and incision models.	Rats	[128]
Propolis	Bahia State, Brazil	Green propolis containing Artepillin C, flavonoids, and other compounds with antibacterial and wound-healing properties.	*Corynebacterium pseudotuberculosis*.	Improved post-surgical healing of caseous lymphadenitis in sheep; reduced wound secretion, enhanced hair regrowth, and inhibited bacterial growth and biofilm formation.	Sheep	[129]
Propolis	El Monofia Province, Egypt	Propolis containing resinous compounds, beeswax, ethereal oils, and bee pollen.	*Enterococcus faecalis* (Not directly tested).	Propolis paste used as intra-canal medicament; effective in promoting regenerative endodontic therapy.	Dogs	[130]
Pollen	Kafrelsheikh, Egypt	Bee pollen containing 62.82% carbohydrates, 19.23% proteins, 4.09% lipids, 2.28% ash, and various amino acids.	Not applicable (growth study).	Bee pollen supplementation improved growth performance, immune system efficiency, and carcass traits.	Broiler chickens	[149]
Bee pollen and propolis	Not specified	Bee pollen rich in flavonoids, carotenoids, and phytosterols.	Not applicable (growth study).	Supplementation with bee pollen and propolis improved growth performance, immune function, and intestinal morphology.	Broiler chickens	[151]
Bee pollen	Saudi Arabia	Bee pollen containing flavonoids, fatty acids, and phytosterols, including quercetin.	*Clostridium difficile*.	Supplementation with bee pollen and propolis improved neuroinflammation and dysbiosis in a rodent model of autism, reducing pro-inflammatory cytokines.	Mice	[152]
Pollen	Dalmatian Coast, Croatia	Bee pollen containing flavonoids such as pinocembrin, chrysin, galangin, isorhamnetin, and quercetin; rich in caffeic acid and kaempferol.	Not tested.	Bee pollen supplementation reduced oxidative stress, improved antioxidant enzyme activity, and. modulated gene expression.	Mice	[154]
Pollen	Kafer El Sheikh, Egypt	Bee pollen containing 21.8% crude protein, 5.2% fat, 2.9% ash, and 67.7% carbohydrates.	Not tested.	Bee pollen and propolis supplementation improved growth performance, fecundity, and semen quality.	Nile tilapia (*Oreochromis niloticus*).	[157]
Royal jelly	Egypt	Royal jelly-containing active proteins such as royalisin, 10-hydroxy-2-decenoic acid, and various fatty acids.	Methicillin-resistant *Staphylococcus aureus* (MRSA).	Emulgel formulation containing 4% royal jelly and 50% garlic extract demonstrated effective wound healing and antibacterial properties against MRSA infections.	Mice	[191]
Jelleine	China	Jelleine-I, a peptide derived from royal jelly, exhibiting antimicrobial, immunomodulatory, and cell-proliferative activities.	*Listeria monocytogenes*.	Effective in inhibiting *Listeria monocytogenes*, reducing biofilm formation, and improving immune responses.	*Galleria mellonella* models	[193]
Royal jelly	South China (Zhejiang)	Royal jelly containing water, proteins, carbohydrates, lipids, vitamins, free amino acids, and minerals.	No bacterial growth was detected.	Royal jelly harvested during *Castanea mollissima* and *Brassica napus* blossom seasons showed distinct wound-healing effects.	Rats	[194]
